# Supplementary material for: Energetic Polymer Possessing Furazan, 1,2,3-Triazole, and Nitramine Subunits
Source: Int J Mol Sci. 2023 Jun 1;24(11):9645. doi: 10.3390/ijms24119645 (PMC10253361; doi:10.3390/ijms24119645)
Supplement: Supplementary file 1 [file ijms-24-09645-s001.zip › ijms-2421375-supplementary.pdf]

International Journal of Molecular Sciences, **2023**

Supporting Information

Energetic polymer possessing furazan, 1,2,3-triazole and nitramine subunits

Pavel S. Gribov,[1] Natalia N. Kondakova,[2] Natalia N. Il'icheva,[2] Evgenia R. Stepanova,[2] Anatoly P. Denisyuk,[2] Vladimir A. Sizov,[2] Varvara D. Dotsenko,[2] Dmitry B. Vinogradov,[1] Pavel V. Bulatov,[1] Valery P. Sinditskii,[2] Kyrill Yu. Suponitsky,[3,4] Mikhail M. Il'in,[3] Mukhamed L. Keshtov,[3] and Aleksei B. Sheremetev[1,]*

[1] *Zelinsky Institute of Organic Chemistry, Russian Academy of Sciences*, 47 Leninsky prosp., Moscow 119991, Russian Federation

[2] *Mendeleev University of Chemical Technology*, 9 Miusskaya pl., Moscow 125047, Russian Federation

[3] *Institute of Organoelement Compounds, Russian Academy of Sciences*, Moscow 119991, Russian Federation

[4] *Basic Department of Chemistry of Innovative Materials and Technologies, Plekhanov Russian University of Economics, 36 Stremyannyi Line, Moscow 117997, Russian Federation*

Table of Contents

X-ray diffraction **2**

Quantum chemical calculation **2**

GPC analysis **5**

Decomposition in isothermal conditions **5**

References **6**

NMR spectra 7

X-ray diffraction

Single crystal X-ray diffraction experiment was carried out using SMART APEX2 CCD diffractometer (λ(Mo-Kα) = 0.71073 Å, graphite monochromator, ω-scans) at 120 K. Collected data were processed by the SAINT and SADABS programs incorporated into the APEX2 program package [73]. The structure was solved by the direct methods and refined by the full-matrix least-squares procedure against F^2 in anisotropic approximation. The refinement was carried out with the SHELXTL program [74]. The CCDC number 2251638 contains the supplementary crystallographic data for this paper. These data can be obtained free of charge via www.ccdc.cam.ac.uk/data_request/cif

Crystallographic data for compound **9**: $C_{10}H_{14}N_4O_6$ are monoclinic, space group $P2_1/c$: a = 9.3983(11) Å, b = 11.1936(13) Å, c = 6.0855(7) Å, β = 103.411(2)°, V = 622.74(13) Å^3, Z = 2, M = 286.25, d_{cryst} = 1.527 g·cm^{-3}. $wR2$ = 0.0823 calculated on F^2_{hkl} for all 1652 independent reflections with $2\theta < 58.0°$, (GOF = 1.079, R = 0.0314 calculated on F_{hkl} for 1566 reflections with $I > 2\sigma(I)$).

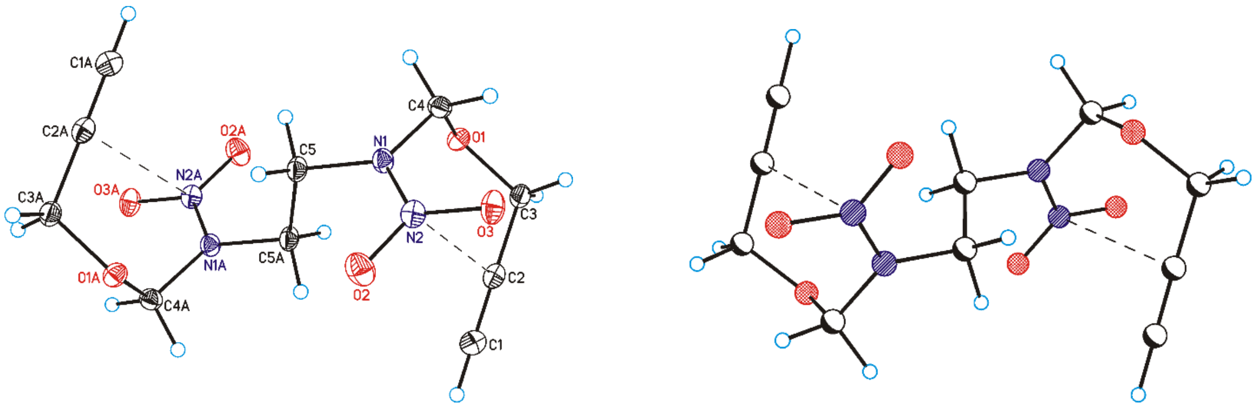

Figure S1. General view of compound **9** (Experimental).

Quantum chemical calculation

Optimization of the geometry of compounds was carried out using the Gaussian program [75] at M052X/def2tzvp level of approximation which was succesfully adopted in our earlier calculations [76-78]. Initial geometry was taken from the X-ray data.

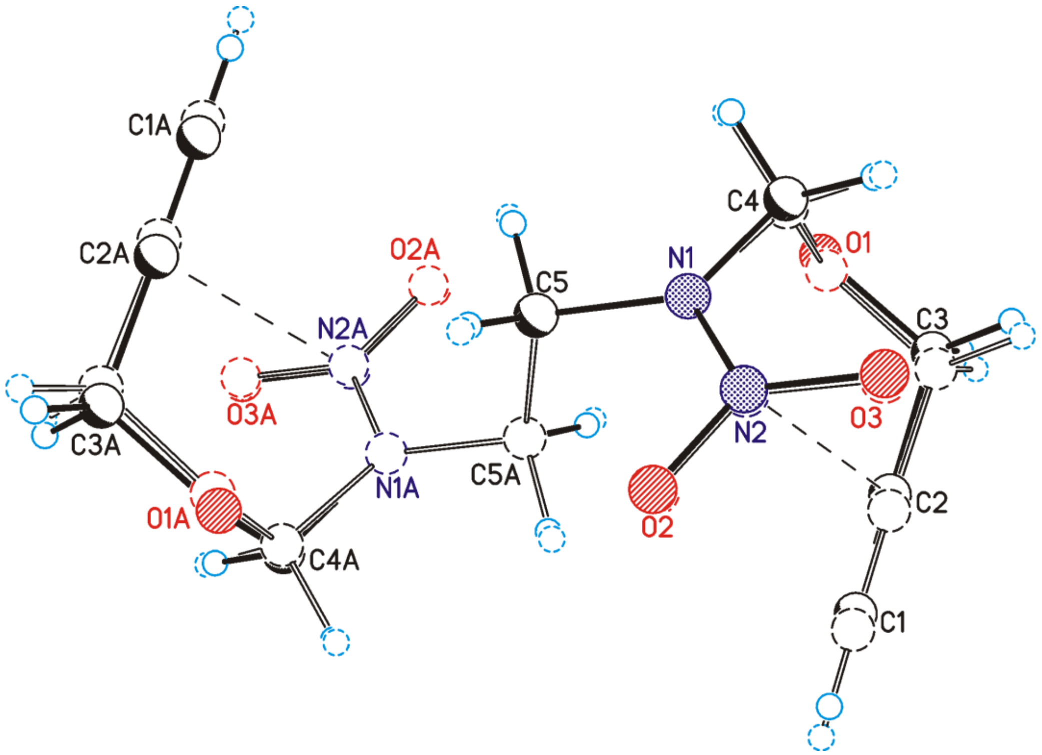

Figure S2. Superimposition of calculated geometry onto experimental one for compound **9**.

Experimental bond lengths and angles are provided in Tables 1S and 2S.

Table S1. Experimental bond lengths for compound **9**.

Bond	Length, Å
O1-C4	1.4111(11)
O1-C3	1.4345(10)
O2-N2	1.2356(10)
O3-N2	1.2347(10)
N1-N2	1.3555(11)
N1-C4	1.4570(11)
N1-C5	1.4596(11)
C1-C2	1.1945(14)
C1-H1	0.95
C2-C3	1.4716(12)
C3-H3A	0.99
C3-H3B	0.99
C4-H4A	0.99
C4-H4B	0.99
C5-C5	1.5351(17)
C5-H5A	0.99
C5-H5B	0.99

Table S2. Experimental bond angles for compound **9**.

Bond angle	Value, °
C4-O1-C3	114.40(7)
N2-N1-C4	120.04(7)
N2-N1-C5	118.20(7)
C4-N1-C5	121.44(7)
O3-N2-O2	123.96(8)
O3-N2-N1	118.42(8)
O2-N2-N1	117.62(8)
C2-C1-H1	180.0
C1-C2-C3	179.31(10)
O1-C3-C2	113.17(7)
O1-C3-H3A	108.9
C2-C3-H3A	108.9
O1-C3-H3B	108.9
C2-C3-H3B	108.9
H3A-C3-H3B	107.8
O1-C4-N1	112.79(7)
O1-C4-H4A	109.0
N1-C4-H4A	109.0
O1-C4-H4B	109.0
N1-C4-H4B	109.0
H4A-C4-H4B	107.8
N1-C5-C5A	111.77(9)
N1-C5-H5A	109.3
C5A-C5-H5A	109.3
N1-C5-H5B	109.3
C5-C5-H5B	109.3
H5A-C5-H5B	107.9

GPC analysis

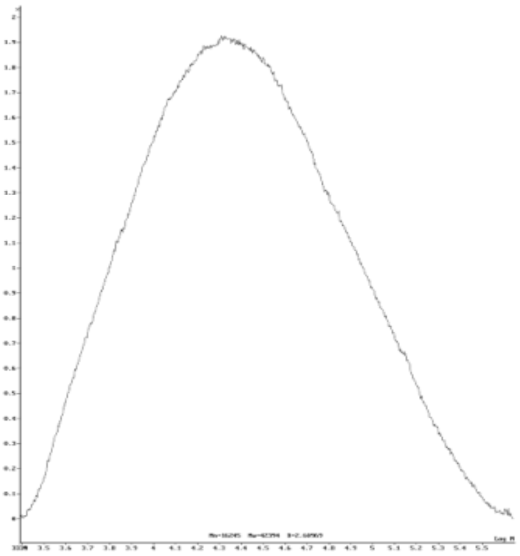

Figure S3. GPC analysis of polymer **12**

Decomposition in isothermal conditions

The experiments on the decomposition of compound **9** under isothermal conditions were carried out in thin-walled glass manometers of the compensation type (the Bourdon glass gauge). Samples of about 20-60 mg weight were loaded into a glass manometer with a volume of 10-12 cm^3. The ampules were vacuumed to 0.1 Torr, sealed, and put in a thermostat with the Wood's alloy. Pressure of gases evolved in the decomposition experiments (the accuracy of pressure measurements was ±1 mm Hg) was converted to the gas volume (V) at normal conditions. Examples of the obtained V *vs*. time curves are shown in Fig S4.

The description of the experimental dependence of gas release on time V(t) by a suitable model allows one to obtain the rate constants. The rate constant of compound **9** was calculated using the model of first-order reaction with self-acceleration:

$$V = V_{end} \cdot k_1 (exp((k_1+k_2) \cdot t)-1)/(k_2 + k_1 \cdot exp((k_1+k_2) \cdot t)),$$

where V_{end} is the final volume of evolved gases, k_1 is rate constant of non-catalytic stage, k_2 is pseudo first-order rate constant of catalytic stage, and t is time.

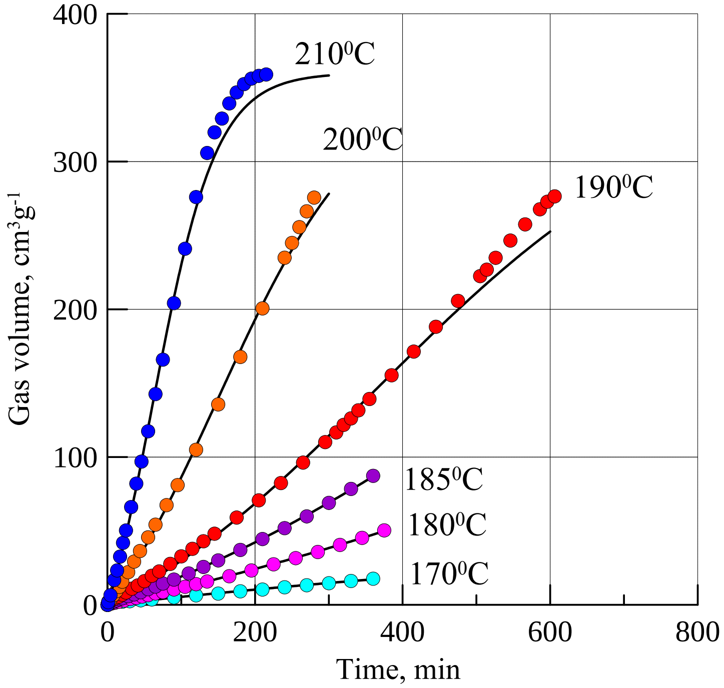

Figure S4. Gas release curves of compound **9** at different temperatures. Points are experiment, lines are fittings

References

73. APEX2 and SAINT; Bruker AXS Inc.: Madison, WI, USA, 2014.
74. Sheldrick, G.M. Crystal structure refinement with SHELXL. *Acta Cryst. C* **2015**, 71, 3-8. DOI: 10.1107/S2053229614024218
75. Frisch, M. J.; Trucks, G. W.; Schlegel, H. B.; Scuseria, G. E.; Robb, M. A.; Cheeseman, J. R.; Montgomery, J. A.; Kudin, K. N., Jr.; Burant, J. C.; Millam, J. M.; *et al*. Gaussian 03, Revision E.01, Gaussian, Inc.: Wallingford, **2004**.
76. Suponitsky, K.Yu.; Smol'yakov, A.F.; Ananyev, I.V.; Khakhalev, A.V.; Gidaspov, A.A.; Sheremetev, A.B. *ChemistrySelect* **2020**, *5*, 14543-14548.
77. Suponitsky, K.Yu.; Fedyanin, I.V.; Karnoukhova, V.A.; Zalomlenkov, V.A.; Gidaspov, A.A.; Bakharev, V.V.; Sheremetev, A.B. *Molecules*, **2021**, *26*, 7452.
78. Suponitsky, K. Yu.; Masunov, A. E.; Antipin, M. Yu. Conformational dependence of the first molecular hyperpolarizability in the computational design of nonlinear optical materials for optical switching. *Mendeleev. Commun.*, **2008**, *18*, 265-267.

^{1}H NMR in DMSO-d6

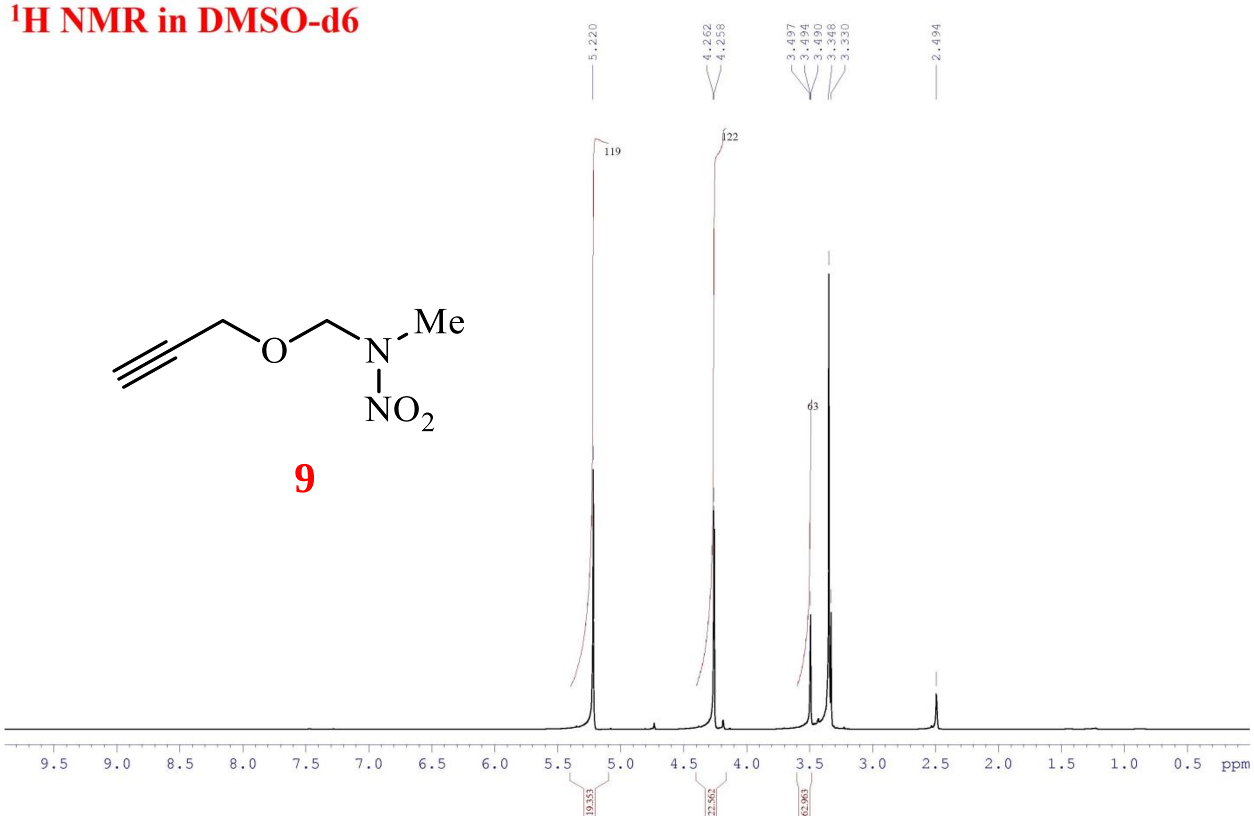

^{13}C NMR in DMSO-d6

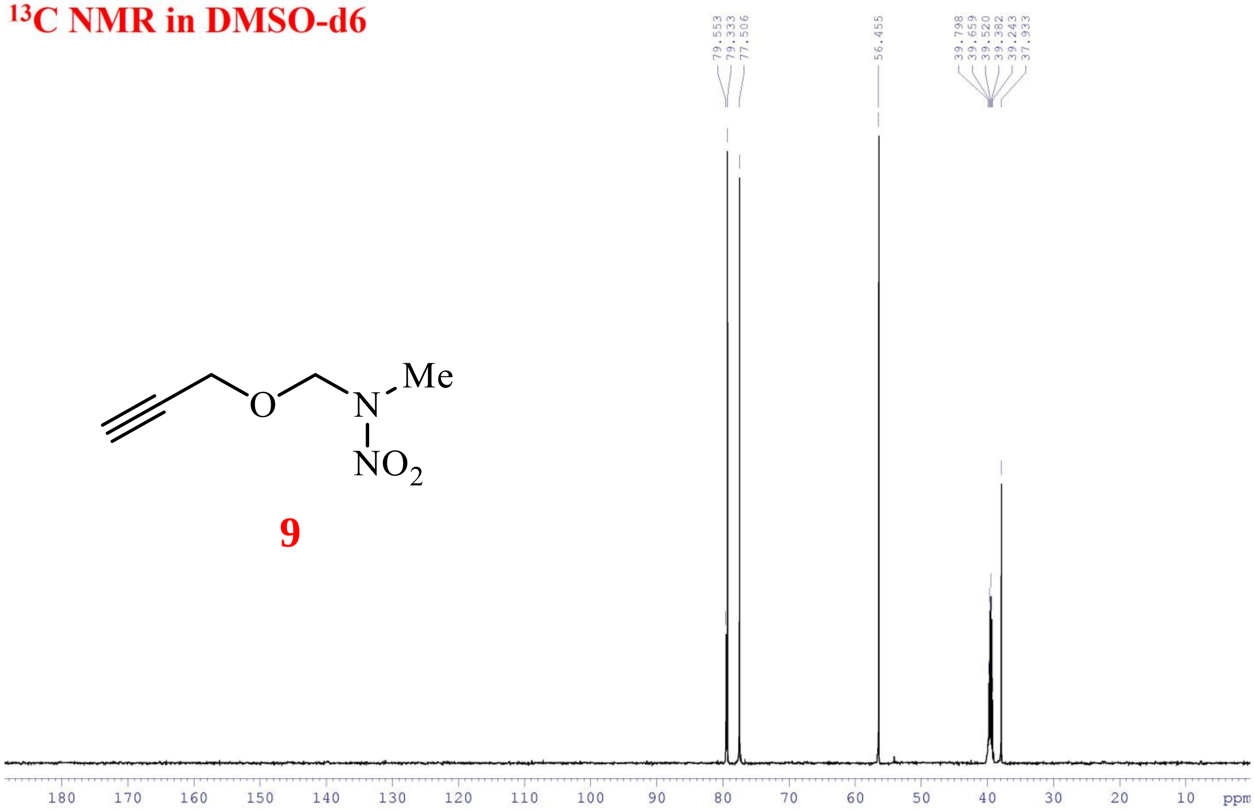

^{14}N NMR in DMSO-d6

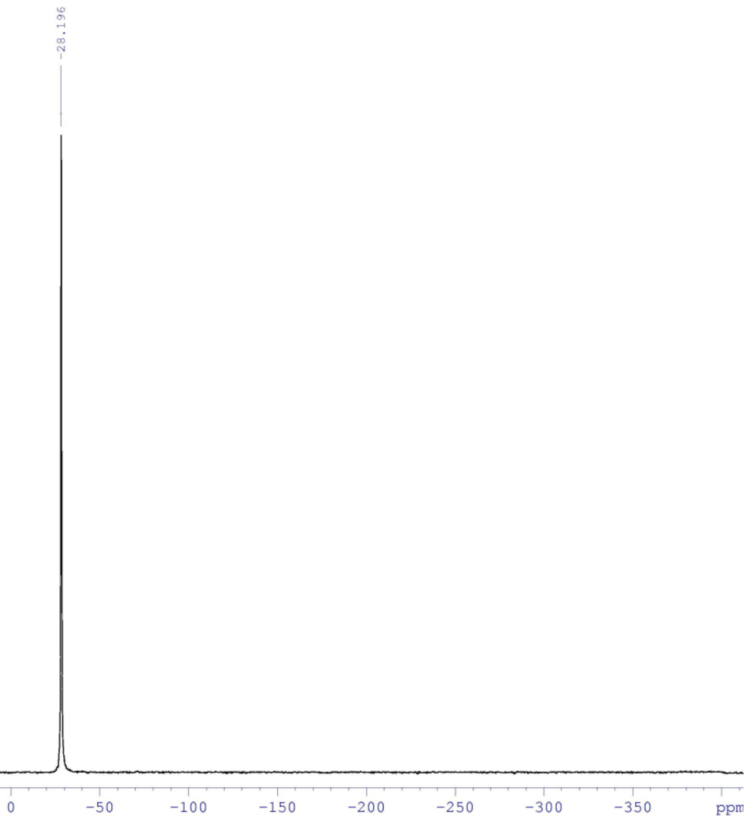

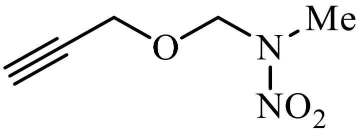

9

^{1}H NMR in DMSO-d6

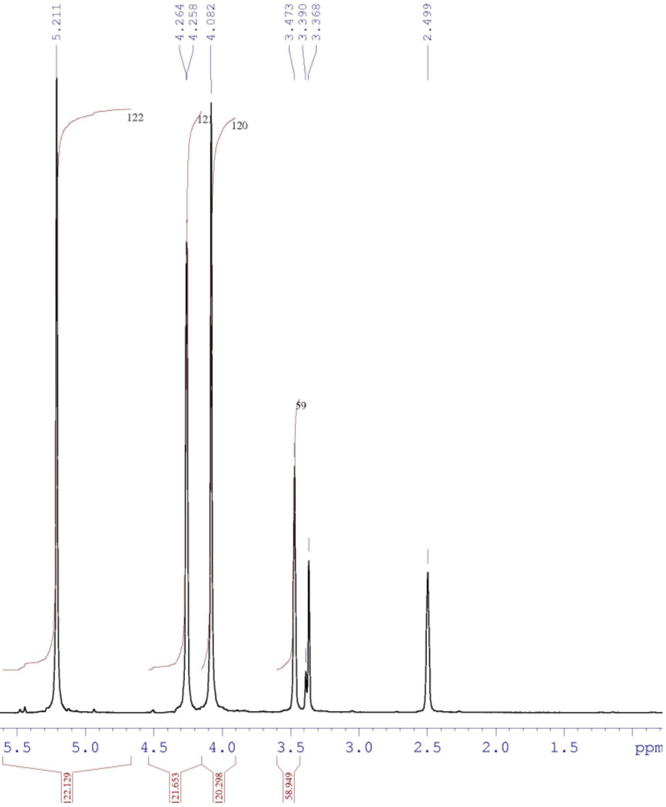

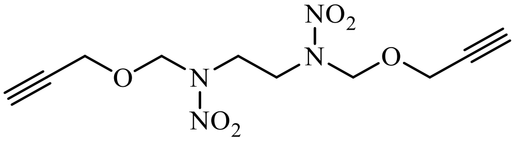

10

^{13}C NMR in DMSO-d6

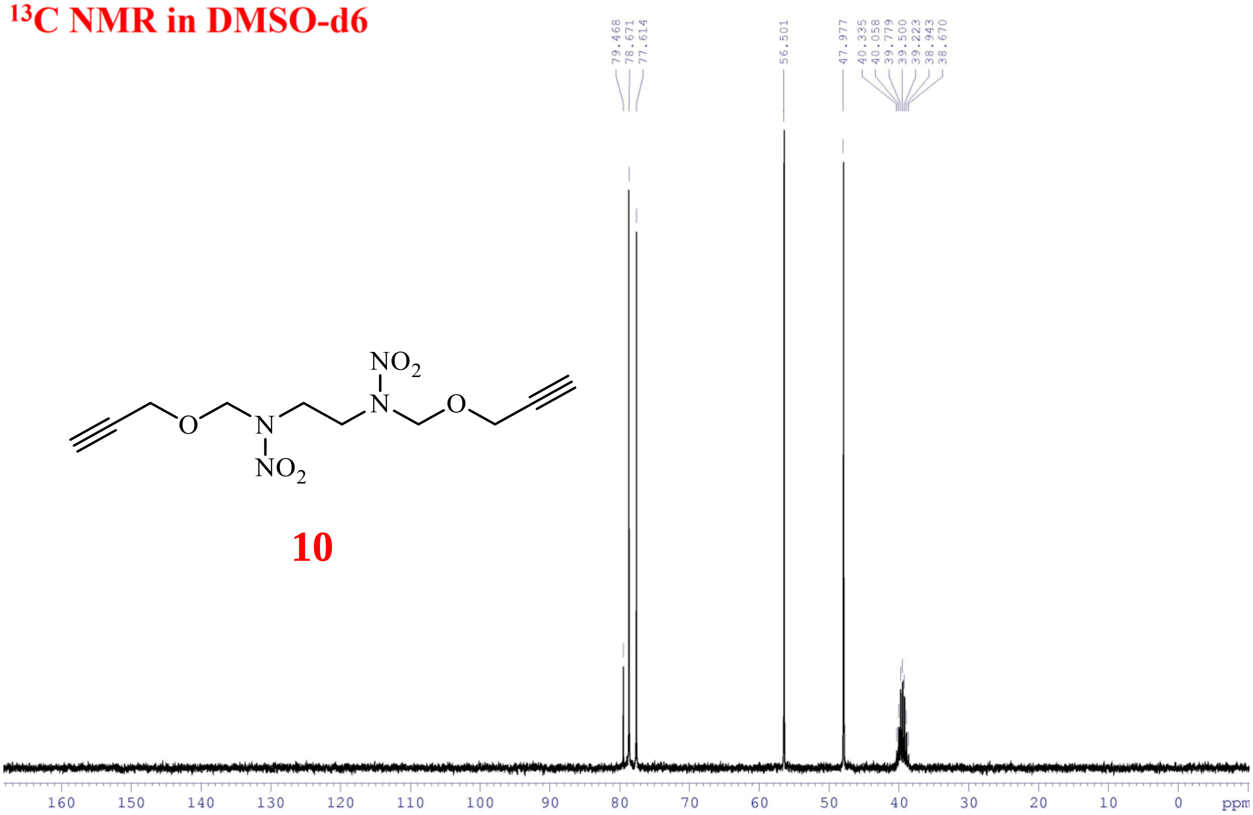

^{14}N NMR in DMSO-d6

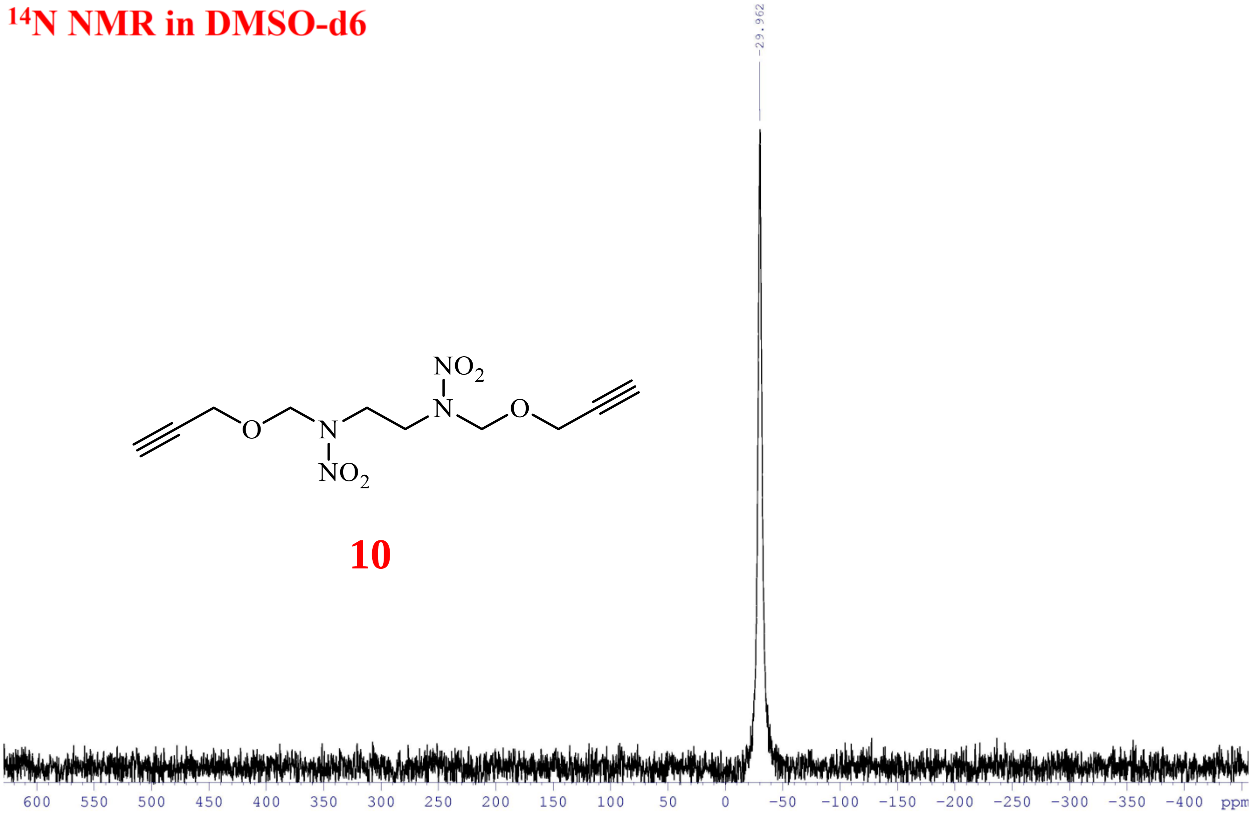

^{1}H NMR in DMSO-d6

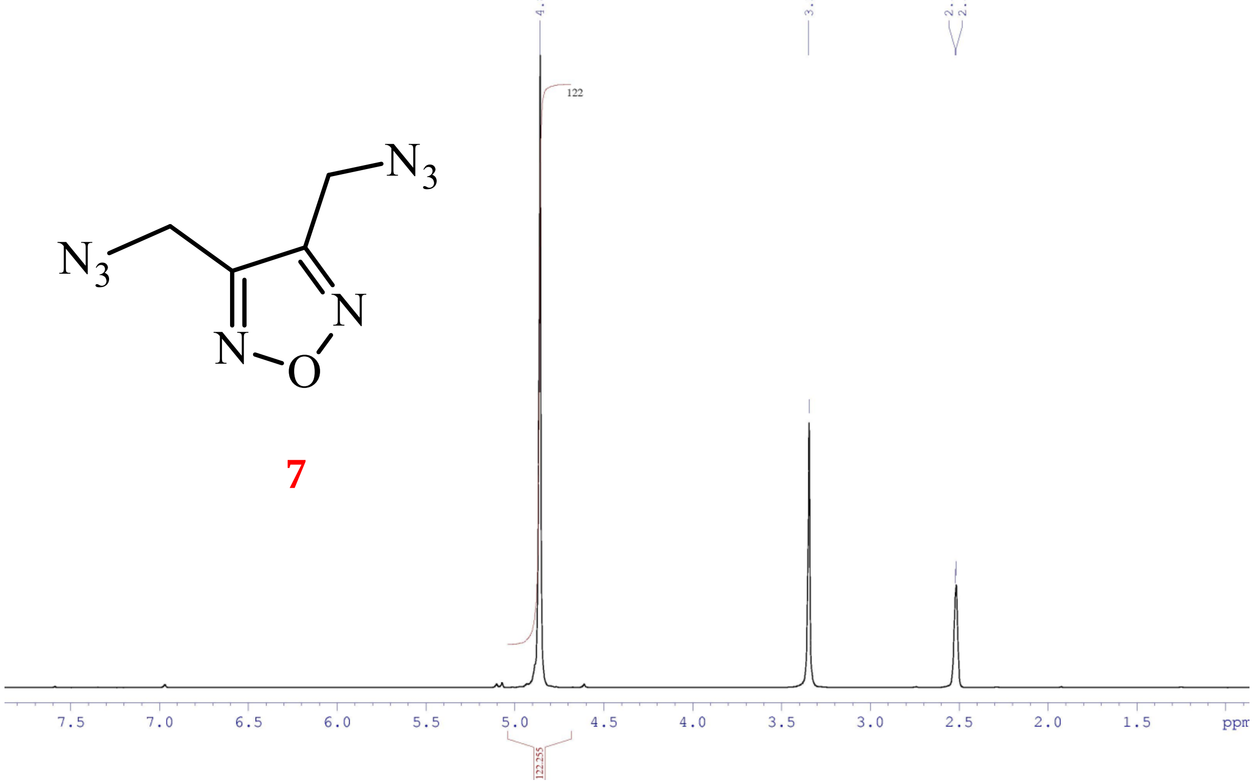

7

^{13}C NMR in DMSO-d6

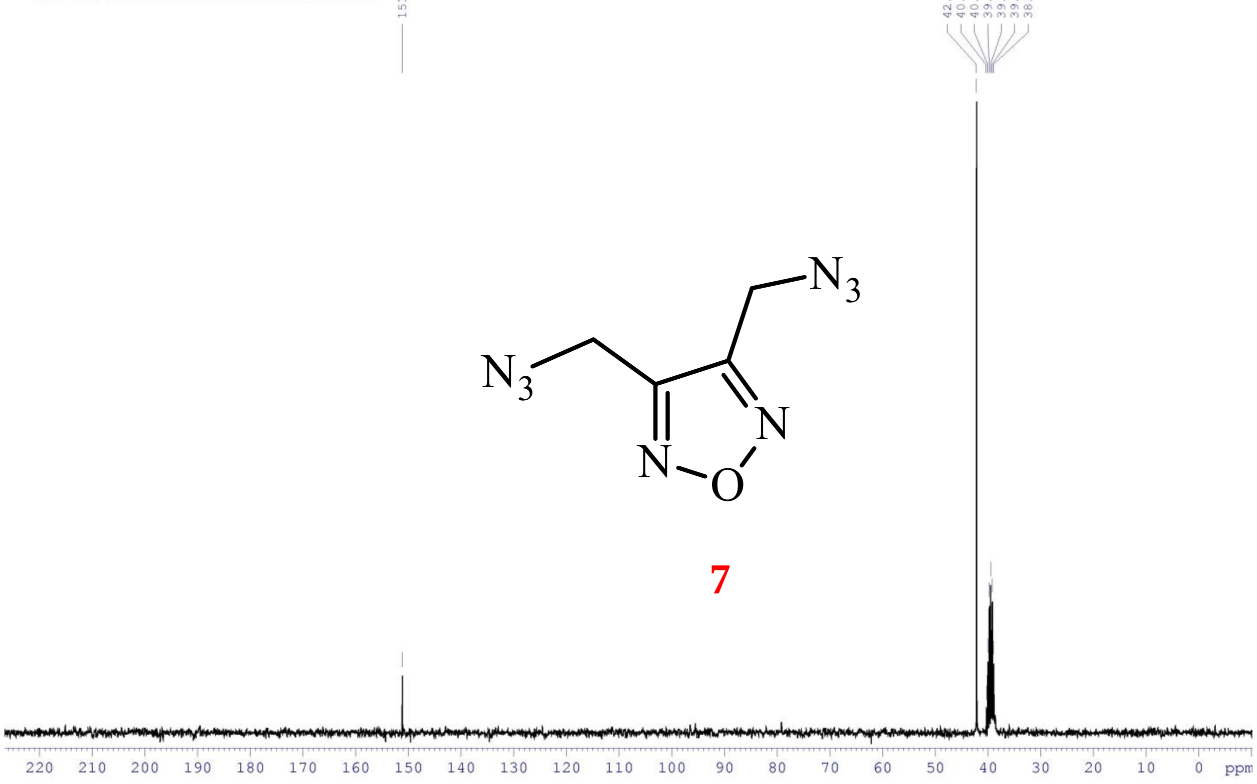

7

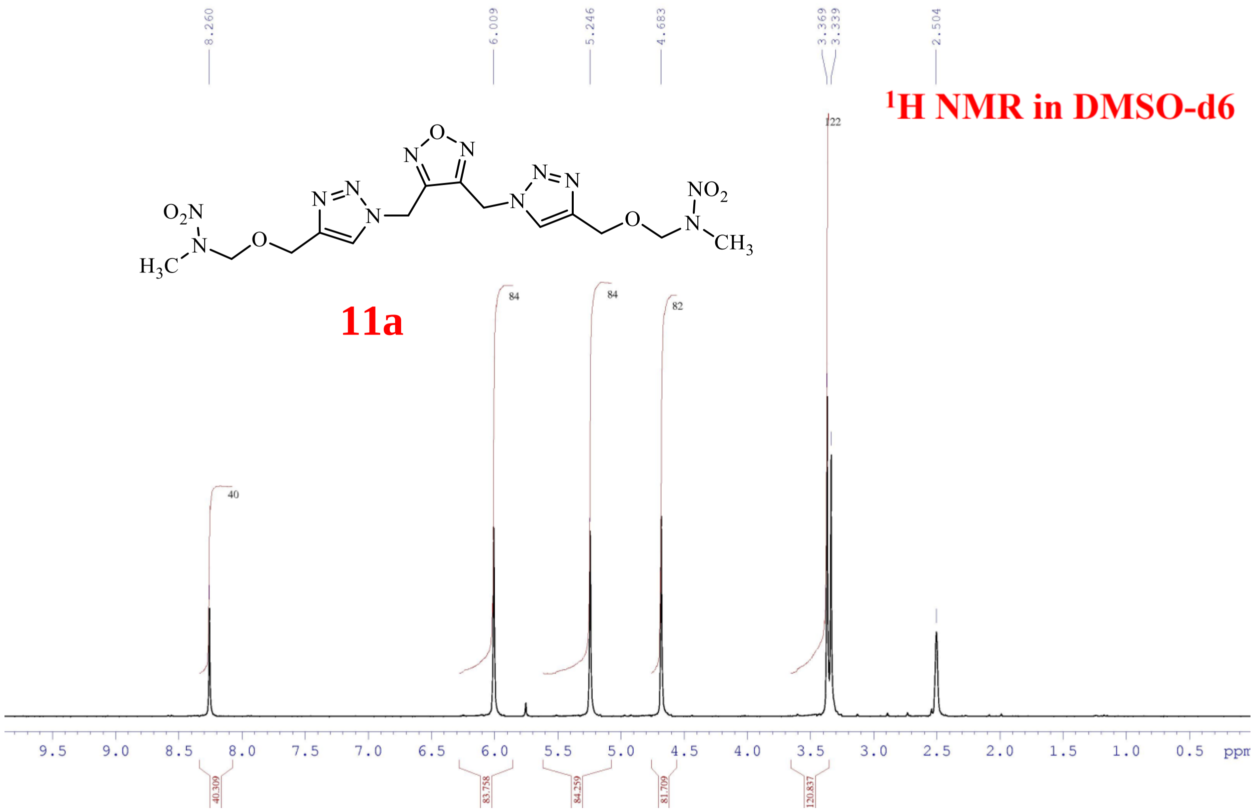

8.260
6.009
5.246
4.683
3.369
3.339
2.504
1H NMR in DMSO-d6
11a
40
84
84
82
122
9.5 9.0 8.5 8.0 7.5 7.0 6.5 6.0 5.5 5.0 4.5 4.0 3.5 3.0 2.5 2.0 1.5 1.0 0.5 ppm
40.309
83.758
84.259
81.709
120.837

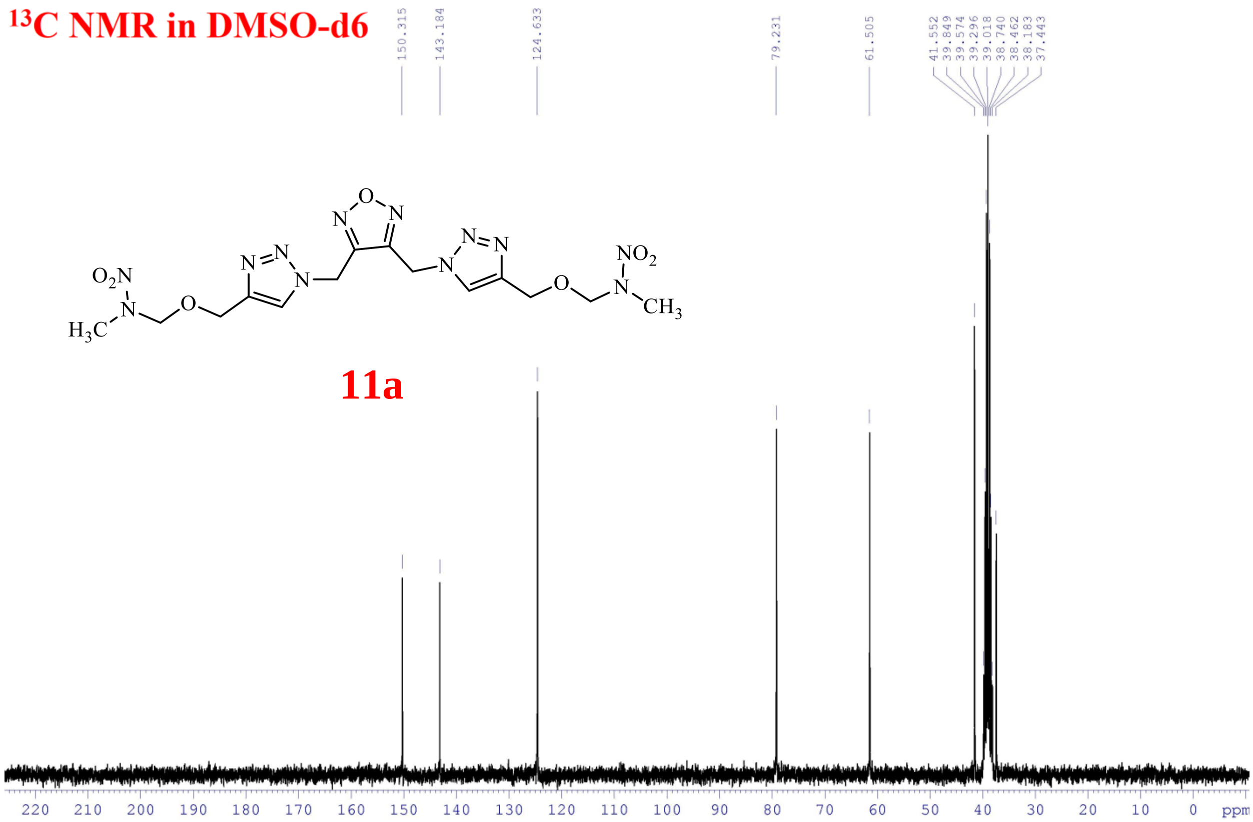

13C NMR in DMSO-d6
150.315
143.184
124.633
79.231
61.505
41.552
39.849
39.574
39.296
39.018
38.740
38.462
38.183
37.443
11a
220 210 200 190 180 170 160 150 140 130 120 110 100 90 80 70 60 50 40 30 20 10 0 ppm

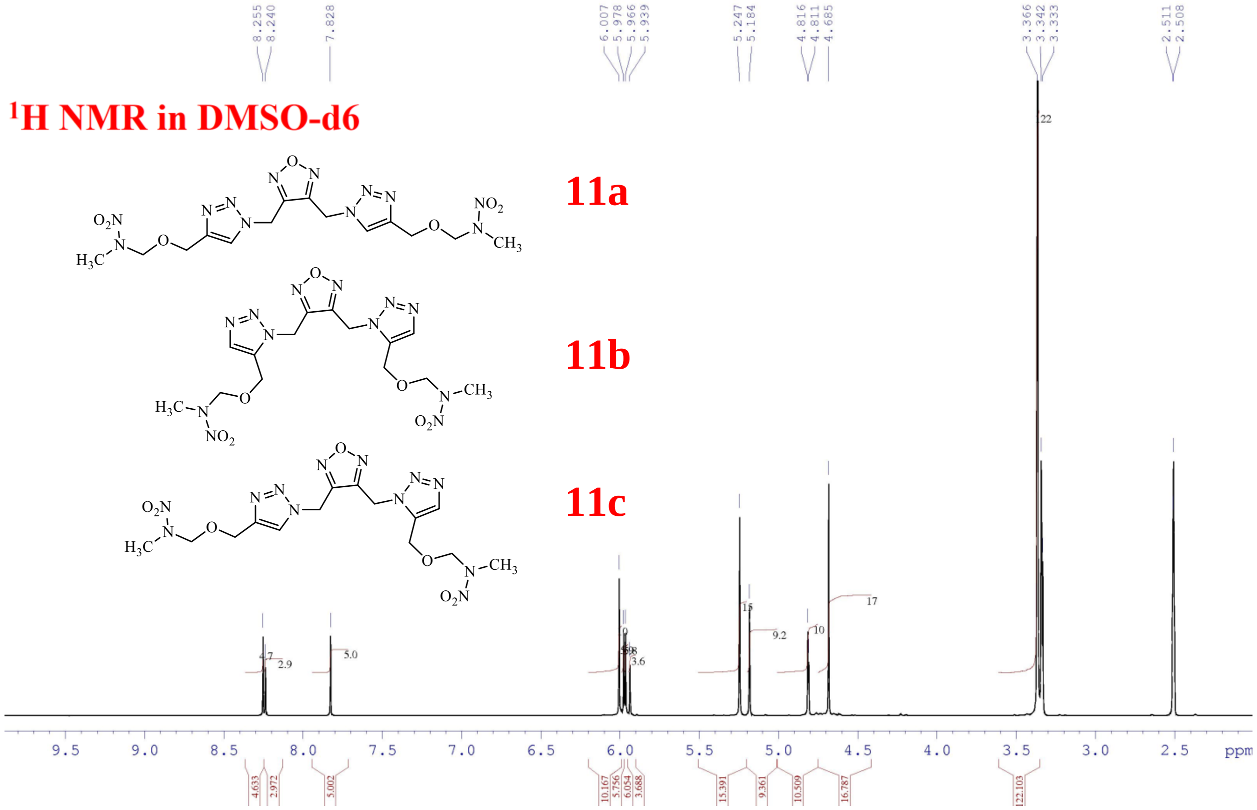

8.255
8.240
7.828
6.007
5.978
5.966
5.939
5.247
5.184
4.816
4.811
4.685
3.366
3.342
3.333
2.511
2.508
^{1}H NMR in DMSO-d6
11a
11b
11c
ppm

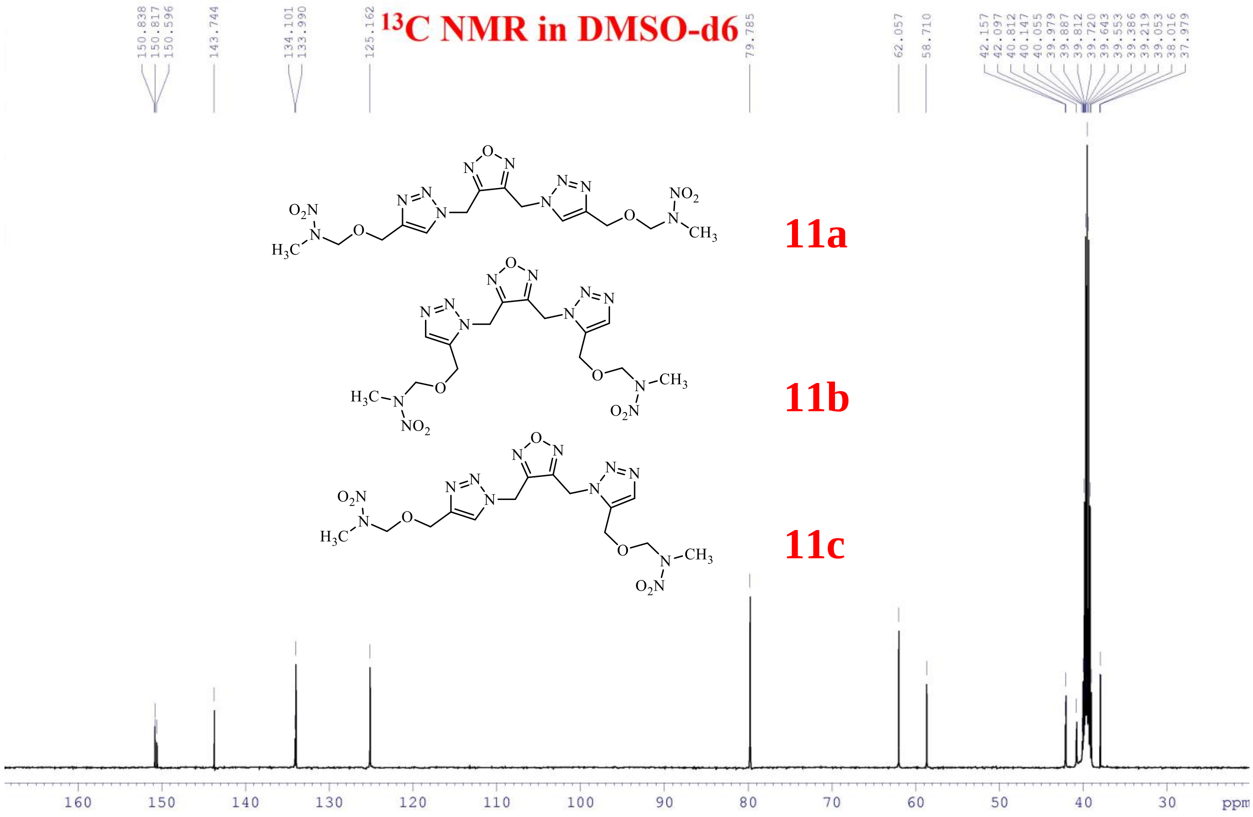

150.838
150.817
150.596
143.744
134.101
133.990
125.162
^{13}C NMR in DMSO-d6
79.785
62.057
58.710
11a
11b
11c
ppm

1H-13C HSQC in DMSO-d6

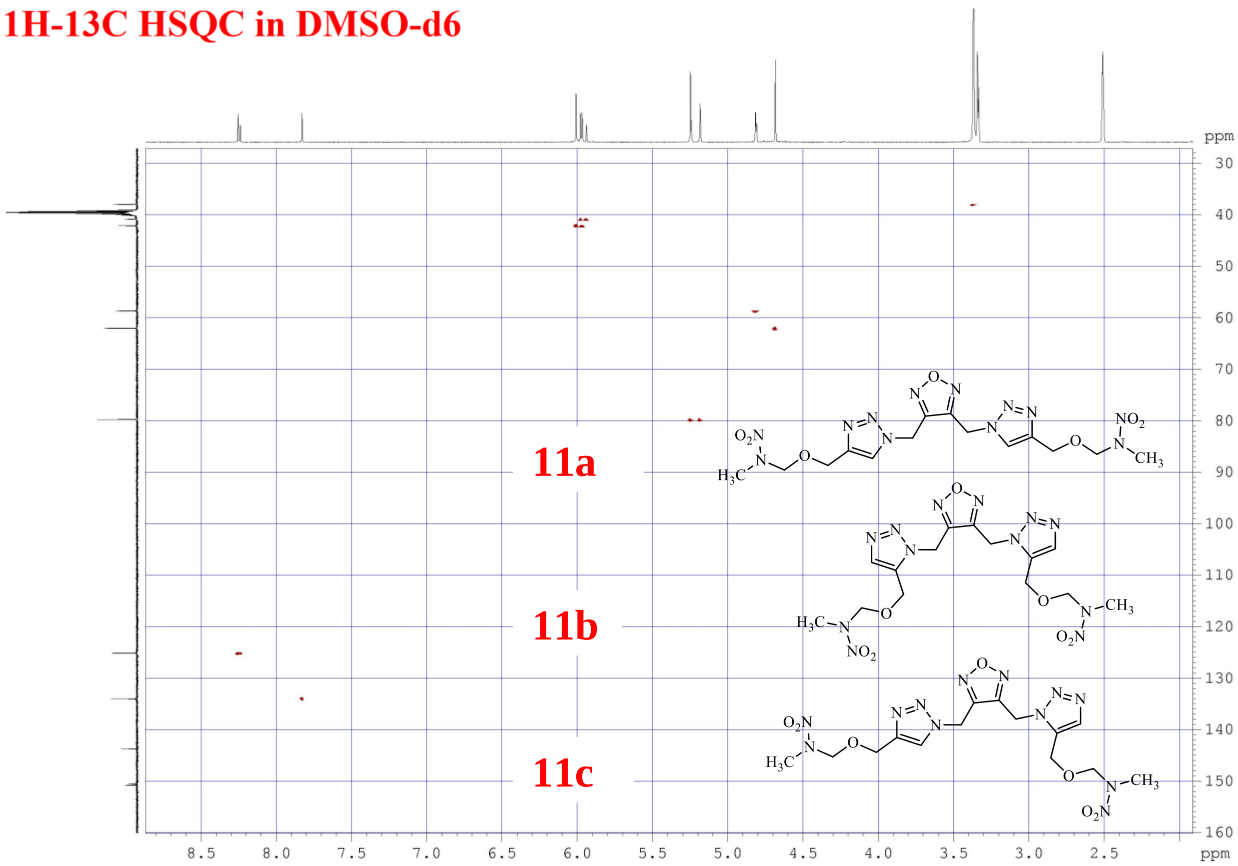

^{1}H NMR in DMSO-d6

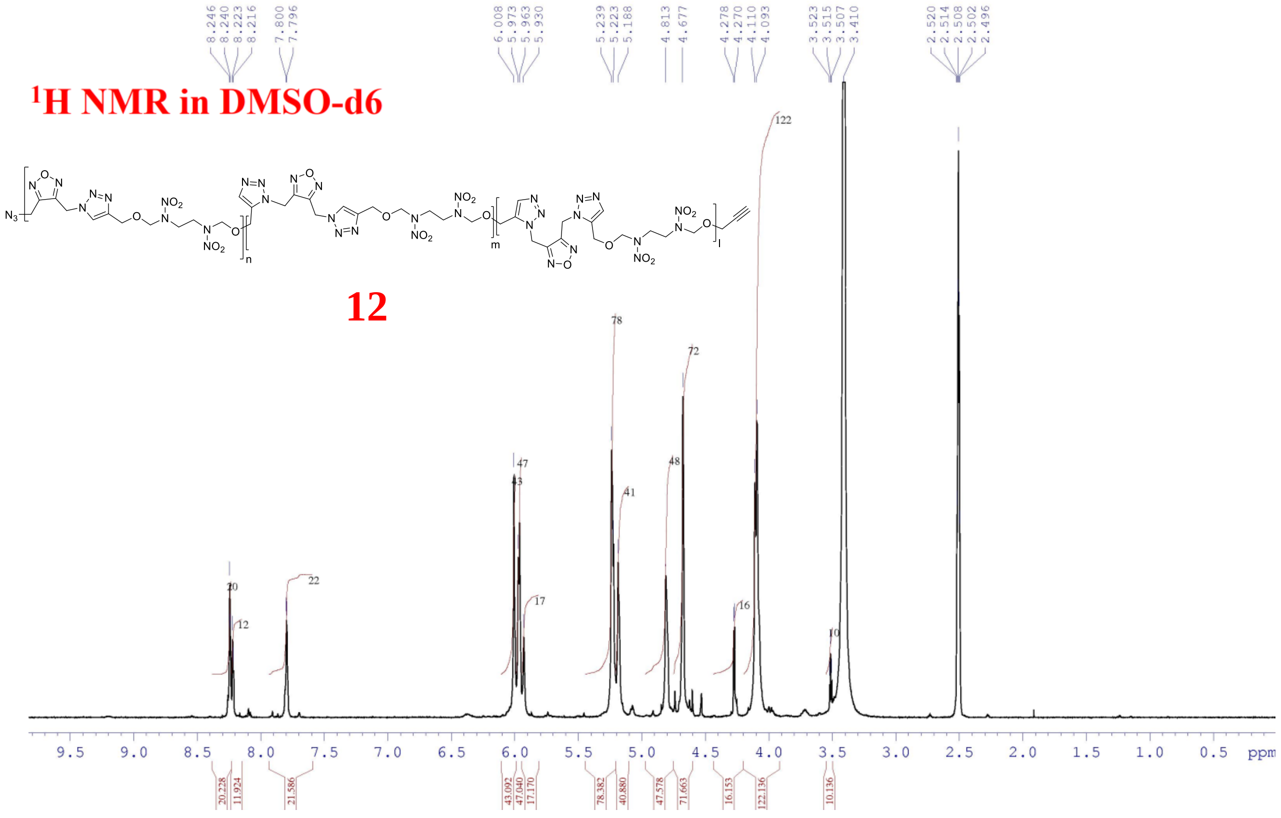

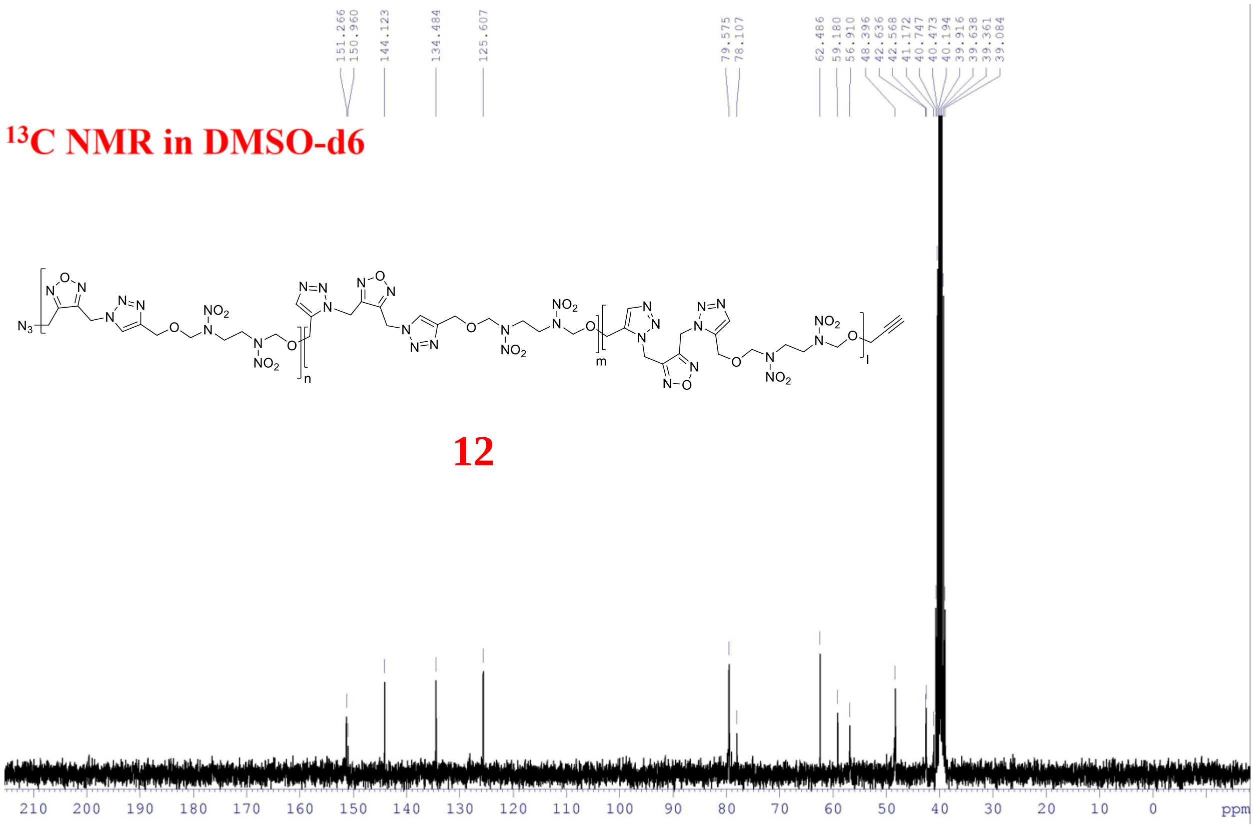

151.266
150.960
144.123
134.484
125.607
79.575
78.107
62.486
59.180
56.910
48.396
42.636
42.568
41.172
40.747
40.473
40.194
39.916
39.638
39.361
39.084
^{13}C NMR in DMSO-d6
12
210 200 190 180 170 160 150 140 130 120 110 100 90 80 70 60 50 40 30 20 10 0 ppm
